# Intestinal microbiome as a diagnostic marker of coronary artery disease: a systematic review and meta-analysis

**DOI:** 10.1097/MS9.0000000000002516

**Published:** 2024-09-04

**Authors:** Yomna E. Dean, Mohamed A. Shebl, Mohamed Doma, Rafeek W. Elmezayen, Jose J. Loayza Pintado, Samah S. Rouzan, Noheir Ashraf Ibrahem Fathy Hassan, Yasmeen E. Yaqout, Akiko Tokunaga, Chukwuebuka Anozie, Omar ElKoumi, Sameh S. Elawady, Tamer Mady, Sana N. Nizam, Yasser Etman, Rayees Nizam, Yusef Hazimeh, Mohamed Alazmy, Hani Aiash

**Affiliations:** a Alexandria University, Faculty of Medicine, Alexandria; b Faculty of Medicine, Cairo University, Kasr Al- Ainy, Cairo; c Kafr-elsheikh University, Kafr-elsheikh; d Aswan University, Faculty of Medicine, Aswan; e Suez Universtiy, Faculty of Medicine, Suez, Egypt; f International American University, College of Medicine, Saint Lucia; g Windsor University School of Medicine, St. Kitts and Nevis, Caribbean; h University for Texas Rio Grande Valley, McAllen, Texas; i Medical University of South Carolina; j SUNY Upstate Medical University, Syracuse; k University of California Los Angeles, California; l Texas Health Hospital Rockwall, Director of Intensive Care Unit, Rockwall, Texas, USA; m Lebanese University; n Zahraa Hospital, University Medical Center, Lebanon; o Ministry of Health, Medical Director, Kuwait; p Sapporo Medical University, Sapporo, Japan

**Keywords:** coronary artery disease, dysbiosis, gastrointestinal microbiome, microbiota

## Abstract

**Background:**

The intestinal microbiome has been recently linked to several metabolic and chronic disorders, one of which is coronary artery disease (CAD). Our study aimed to analyze the intestinal microbiome of CAD patients and assess the eligibility of dysbiosis as a diagnostic marker of CAD.

**Methods:**

PubMed, Scopus, Embase, and Web of Science were searched using terms, such as ‘CAD’ and ‘microbiome’. Only observational controlled studies were included. R version 4.2.2 was used for the analysis.

**Results:**

A significant association was found between the CAD group and increased Simpson and Shannon Indices compared with the control group (MD=0.04, 95% CI=0.03–0.05, and MD=0.11, 95% CI=0.01–0.22, respectively). Our analysis yielded a statistically significant association between the CAD group and increased Prevotella genus (MD=13.27, 95% CI=4.12–22.42, *P*-value=0.004), Catenibacterium genus (MD=0.09, 95% CI=0.09–0.10), Pseudomonas genus (MD=0.54, 95% CI=0.29–0.78, *P*-value), and Subdoligranulum (MD=−0.06, 95% CI=−0.06 to −0.06) compared with the control group. Another significant association was detected between the CAD group and decreased *Bacteroides vulgatus* and *Bacteroides dorei* (MD=−10.31, 95% CI=−14.78 to −5.84, *P*-value <0.00001).

**Conclusion:**

Dysbiosis is an acceptable diagnostic marker of CAD. Decreased *B. dorei* and *B. vulgatus* among CAD patients suggests a protective role of these bacteria. Future clinical trials are necessary to investigate the potential benefit of supplementation of these bacteria in treating or preventing CAD.

## Introduction

Highlights

- Dysbiosis is an acceptable diagnostic marker of CAD.
- Decreased *Bacteroides dorei* and *Bacteroides vulgatus* among CAD patients suggests a protective role of these bacteria.
- Future clinical trials are necessary to investigate the benefit of their supplementation in treating or preventing CAD.

Coronary artery disease (CAD) is the most common heart disease and is the leading cause of death in the United States^1,2^. Recently, advancements in diagnostic modalities for coronary artery disease (CAD) have expanded to explore novel techniques for various heart conditions similar to aortic dissection^3^. These techniques range from invasive, noninvasive imaging modalities as well as innovative diagnostic markers such as intestinal microbiota which has recently emerged as the noninvasive technique used as a marker for a wide variety of different diseases, which includes tumors and autoimmune diseases^4^.

The intestinal microbiota is composed of bacteria, fungi, archaea, and viruses that inhabit the gastrointestinal tract forming a heterogenous ecosystem that interacts together and with the host intricately. Gut microbiota could be formed of more than 10^14^ microorganisms^5^. Among all these microorganisms, there are four main categories representing most intestinal microbiota including firmicutes, bacteroidetes, actinomycetota, and proteobacteria^6^. Healthy microbiota plays an essential role in human health from protecting against life-threatening infections such as clostridium difficile^7^ to the synthesis of vitamins such as vitamin K^8^. These functions are essential for the normal physiological function of the human body. Additionally, they have been found to cause an inflammatory state, secrete endotoxins, and/or organic compounds known to cause cardiovascular disease (CVD) or predispose to CVD through causing other disorders (such as diabetes)^9–11^. Furthermore, it was shown that atherosclerotic plaques contained bacterial DNA^12^.

According to recent updates in the literature, there may be a higher risk of coronary artery disease (CAD) due to a lesser variety of the gut microbiome and a different bacterial composition. This implies that gut flora may contribute to the development of CAD, opening new possibilities for probiotics and prebiotics as preventative and therapeutic approaches^13–15^. Studies have also recommended exploration of the role of the microbiome in blood pressure regulation^16^.

The aim of our study is to perform a meta-analysis to determine whether different gut microbiota genres are useful as CAD diagnostic markers.

## Methods

### Search strategy and literature search

**Figure 1:**
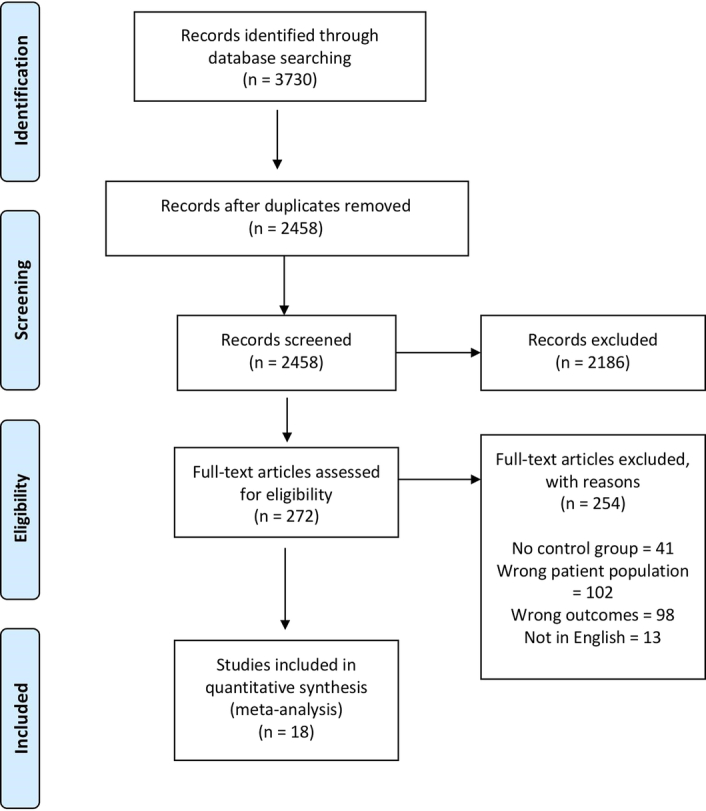
PRISMA.

PubMed, Scopus, Embase, and Web of Science were searched on the 6th of May 2022, using key terms such as ‘Microbiome’, ‘dysbiosis’, ‘intestinal bacteria’, ‘coronary artery disease’, ‘angina’, and ‘acute coronary syndrome’ (View the supplementary material for the full search strategy, Supplemental Digital Content 1, http://links.lww.com/MS9/A595). This yielded 3730 studies and after the removal of duplicates, the number was reduced to 2458. Of these, 272 were eligible for full‐text screening. Finally, 18 studies were included in the meta‐analysis^17–34^, as shown in the Prisma flowchart (Fig. 1).

### Inclusion and exclusion criteria

We screened studies by titles and abstracts according to the following criteria:

Inclusion criteria: controlled observational studies reporting data on the intestinal microbiome among adults (≥18 years old) suffering from CAD, including cross-sectional, case–control, and cohort studies.

Exclusion criteria: editorials, commentaries, reviews, systematic reviews, meta-analyses, case reports, case series, animal studies, and studies lacking a control group.

In the case of duplicate studies, the most recent study with the largest study population was included.

### Study selection

Two independent reviewers (M.S. and Y.D.) screened the titles and abstracts of the studies according to our criteria. If a consensus is not achieved, a third independent reviewer was assigned to resolve the conflict.

### Data extraction

Two authors (M.S. and S.R.) independently extracted each study. The data were then compared to confirm accuracy. If a consensus was not achieved, a third independent reviewer was assigned to resolve the conflict.

For the baseline and summary, the following data were extracted: last name of the first author, year of publication, study design, country, sample type, sample size, sex, BMI, smoking, dyslipidemia, the prevalence of hypertension, diabetes, and conclusion.

For the outcomes, the following data were extracted: diversity indices, the relative abundance of different phyla, order, genus, species, and the area under the curve for the sensitivity analysis.

### Quality assessment

Two independent authors conducted the risk of bias assessment (M.S. and S.H.). Disagreements were resolved through consensus after discussing reasons for the discrepancies. Studies were appraised with the Newcastle–Ottawa Scale (NOS). In this scale, studies are scored on a 0 to 9 scale according to the quality of patient selection, comparability of groups and adjudication of outcomes. We defined the observational studies with a NOS score of ≥7 stars as high-quality and NOS score of <7 stars as low quality^35^. Publication bias was investigated by funnel-plot analysis of point estimates according to study weights and by Egger’s regression test for outcomes including 10 studies or more.

### Statistical analysis

We summarized binary endpoints using the Mantel–Haenszel random-effects model, with risk ratio (RR) and 95% CI as a measure of effect size. The Der Simonian and Laird (DL) method was used to calculate heterogeneity variance τ². Heterogeneity was assessed with Cochrane’s Jackson method’s *I*² statistics, with *P*≤0.10 indicating statistical significance. The consistency of the studies was determined based on *I*² values of 0%, ≤25%, ≤50%, and >50%, indicating no observed, low, moderate, and substantial heterogeneity, respectively. All tests were two-tailed, and a *P*-value of <0.05 was considered statistically significant. We used R version 4.2.2 (R Foundation for Statistical Computing) and the extension packages ‘meta’ and ‘dmetar’ for all calculations and graphics^36–38^.

### Subgroup and meta-regression analysis

We performed a prespecified subgroup analysis for abundance of gut microbiome according to different bacterial phylum, class, genus, and species (Figs 2–12).

**Figure 2:**
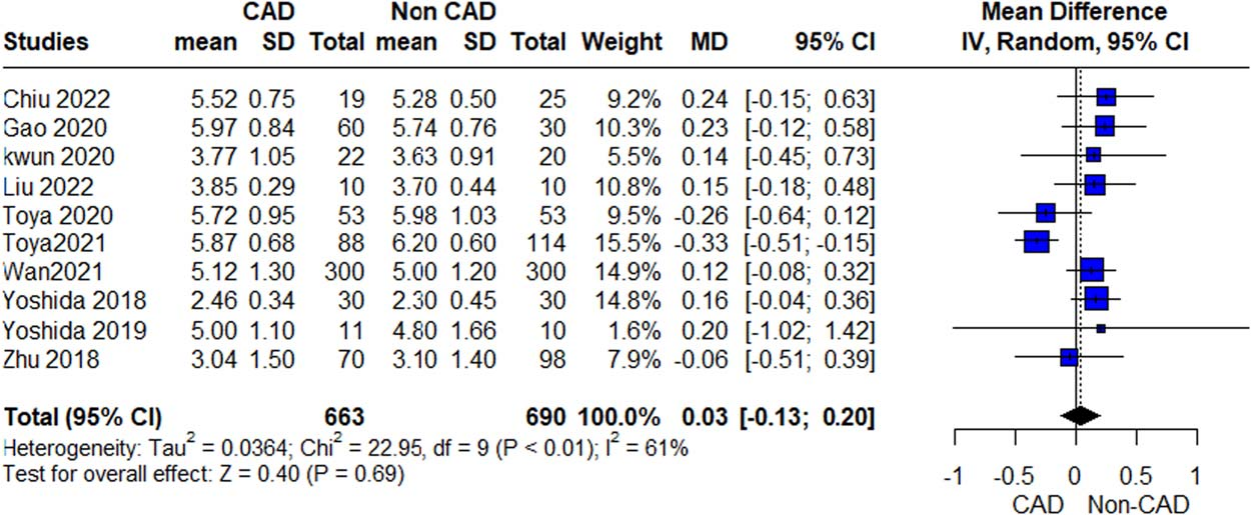
Shannon Index outcome.

**Figure 3:**
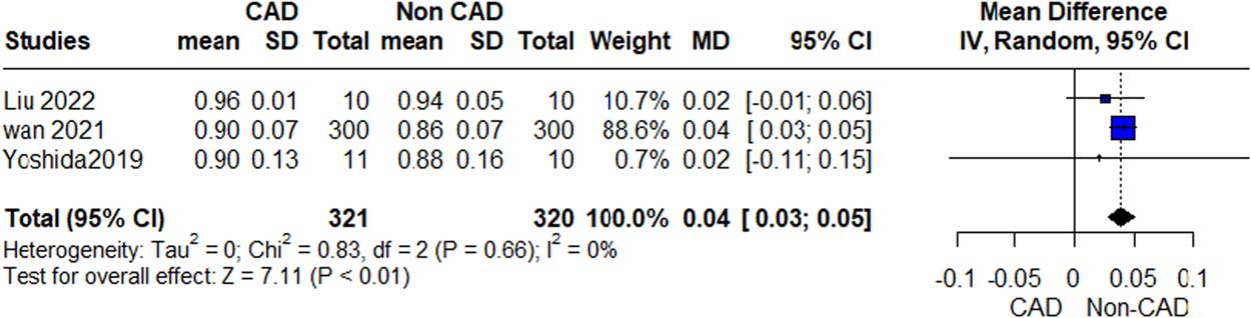
Simpson Index outcome.

**Figure 4:**
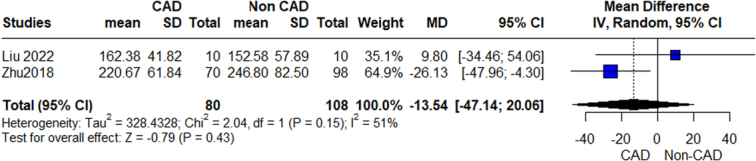
Relative abundance phylum firmicutes outcome.

**Figure 5:**
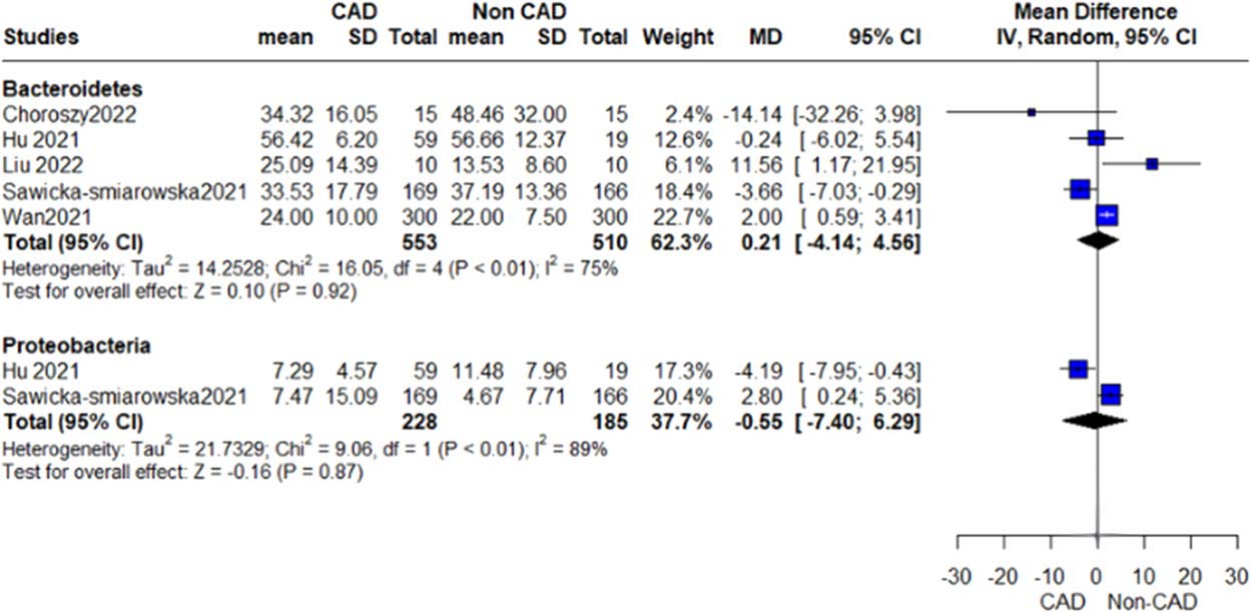
Relative abundance phylum outcome.

**Figure 6:**
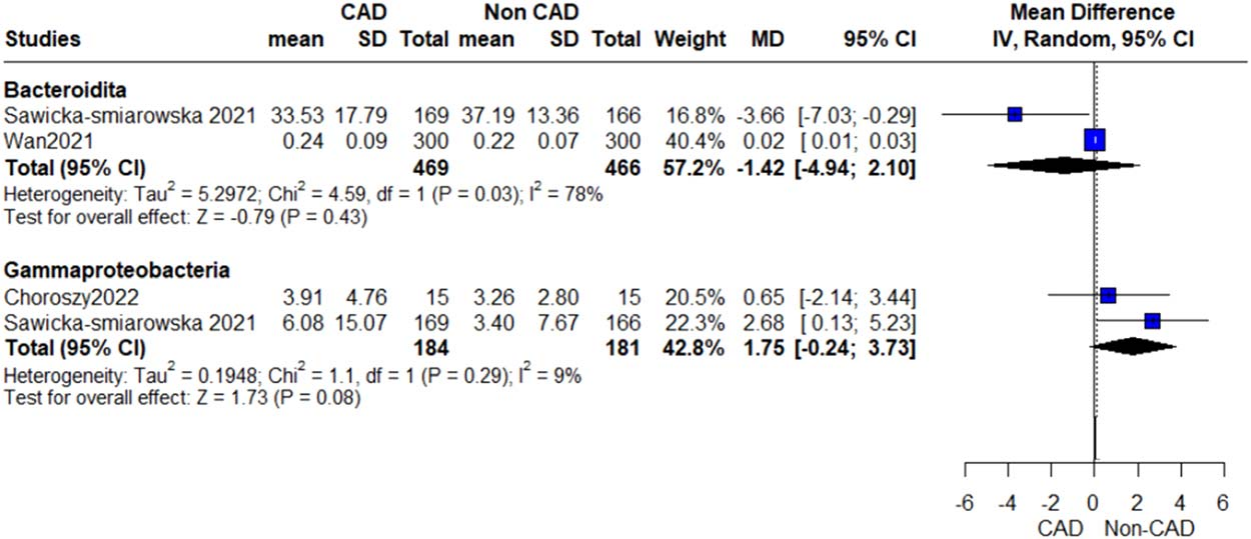
Relative abundance class outcome.

**Figure 7:**
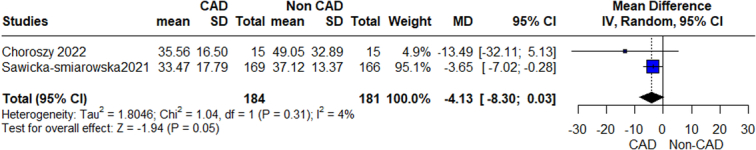
Relative abundance order bacteroidales outcome.

**Figure 8:**
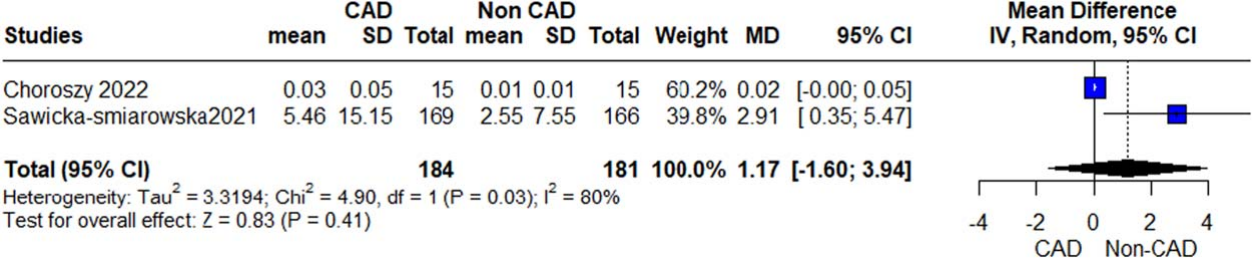
Relative abundance order enterobacteriales outcome.

**Figure 9:**
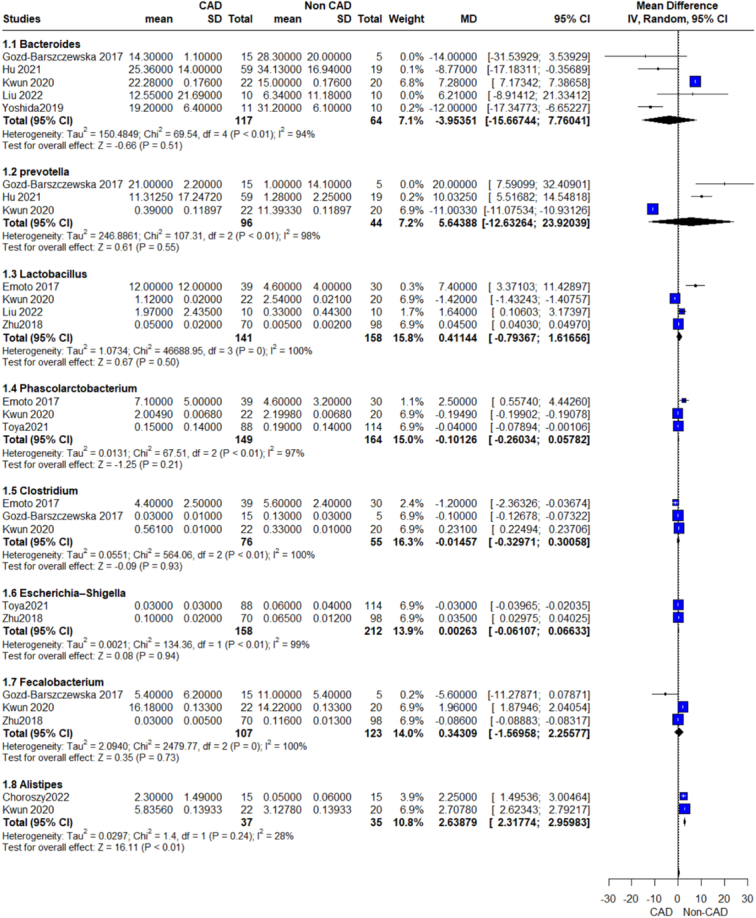
Relative abundance genus outcome.

**Figure 10:**
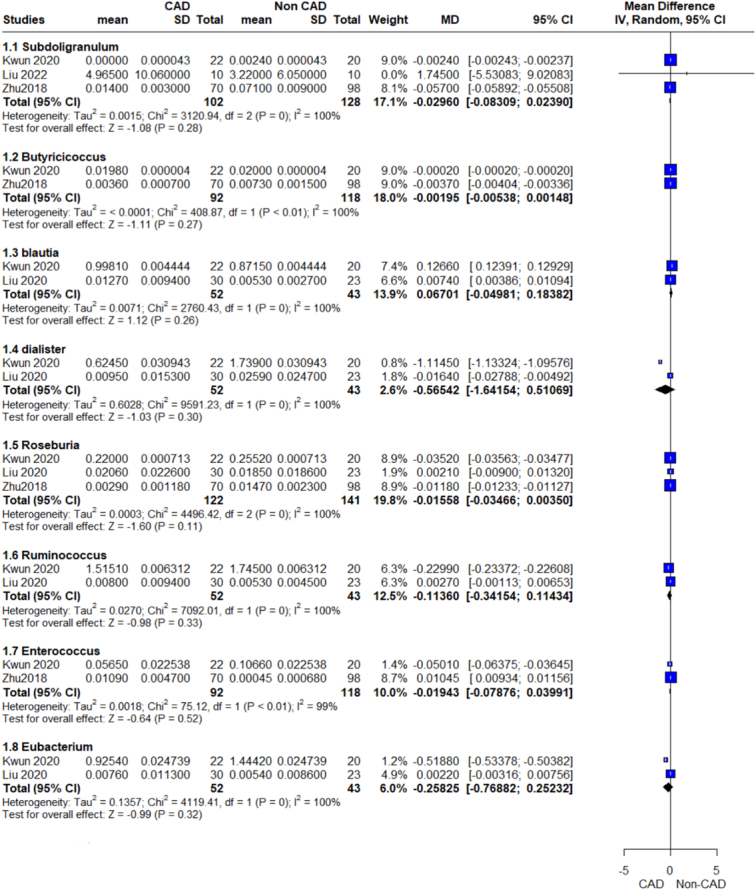
Relative abundance genus outcome.

**Figure 11:**
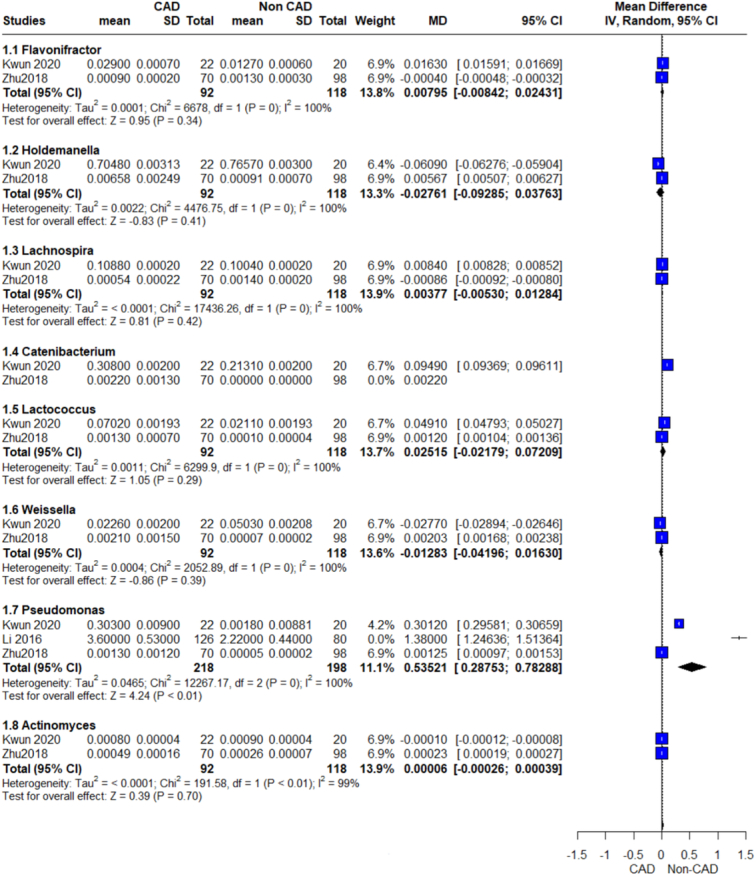
Relative abundance genus outcome.

**Figure 12:**
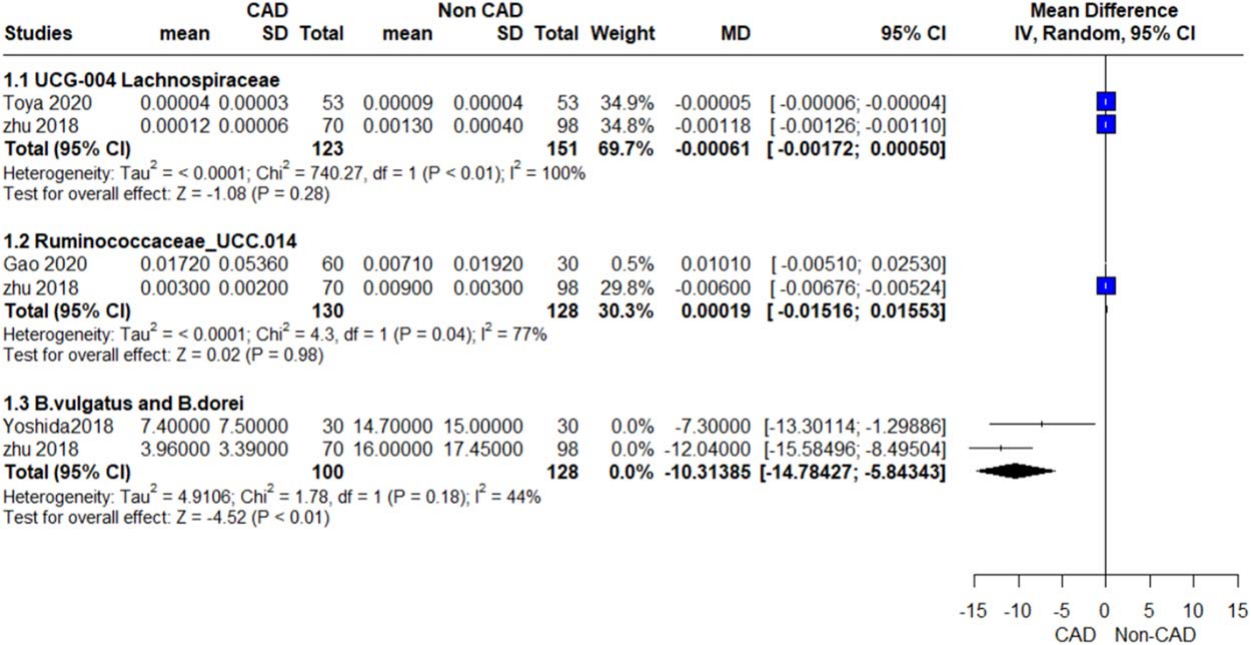
Relative abundance species outcome.

We also performed a meta-regression analysis correlating covariates such as BMI, hypertension, diabetes and smoking for diversity indices outcomes (Supplementary Table S2, Supplemental Digital Content 2, http://links.lww.com/MS9/A596 and Supplementary Fig. S2, Supplemental Digital Content 2, http://links.lww.com/MS9/A596).

## Results

### Characteristics of included studies

This study comprised 2101 patients of whom 1268 had CAD. A complete appraisal of baseline characteristics of the studies included in our analysis is reported in Table 1.

**Table 1:** Baseline characteristics of included studies.

References	Study design	Sample size (CAD/non-CAD)	Sample type	Males, *n* (%)	BMI, mean±SD (CAD/non-CAD)	Smoking, n (%) (CAD/non-CAD)	Hypertension, *n* (%)	Diabetes, *n* (%)	Hyperlipidemia/Dyslipidemia, *n* (%) (CAD/Non-CAD)	Conclusion
Yoshida^32^	Prospective cohort	21 (11/10)	Stool	17 (80.9%)	24±3.1 / 24.3±4.2	6(54)/9 (90)	14 (66.7%)	7 (33.3%)	11(100)/5(50)	Gut microbial analysis showed significantly lower levels of B. vulgatus and B. dorei in the original fecal samples from patients with CAD
Zheng^33^	Prospective cohort	257 (152/105)	Blood and Stool	163 (63.4%)	—	74(49)/35(33.3)	120 (46.7%)	35 (13.6%)		Fecal microbial diversity was increased in CAD patients compared to that in healthy controls. Phylum Bacteroidetes was increased in CAD patients versus healthy controls
Zhu^34^	Case–control	168 (70/98)	Stool	71 (42.3%)	24.34/23.83^a^	12(17.1)/20(20.4)	101 (60.1%)	—	—	Dysbiosis has a role in pathogenesis of CAD. Healthy microbiota is a novel tool for management of Cardiovascular diseases
Chiu^17^	Case–control	44 (19/25)	Stool	—	—	—	29 (65.9%)	7 (15.9%)	5(26)/6(24)	The study demonstrated a decreasing abundance of *Selenomonadales* and an increasing abundance of seleno-compounds after AMI. The findings link gut microbiome and metabolites to AMI
Choroszy 2022	Case–control	30 (15/15)	Stool	16 (53.3%)	29.4±5.05 / 25.95±4.91	—	—	—	—	16S rRNA sequencing analysis revealed gut dysbiosis in CAD patients that was further confirmed by elevated levels of bacterial metabolites
Emoto 2017	Case–control	69 (39/30)	Blood and Stool	56 (81.1%)	25.7±4.1 / 25.6±4.1	34(88)/17(57)	59 (85.5%)	27 (39.1%)	37(90)/20(63)	Gut microbiota may have a potential to be a diagnostic marker of CAD
Gao^20^	Case–control	90 (60/30)	Blood and Stool	70 (77.7%)	25.48±2.42 / 24.39±2.86	—	—	—	—	Results of the present study demonstrated that the gut microbial taxa of ACS patients are clearly different from those of subjects without ACS
Gozd-Barszczewska^21^	Cohort study	20 (15/5)	Blood and Stool	20 (100%)	—	—	—	—	—	Intestinal microbiome is likely to play a role in the pathogenesis of atherosclerosis through its role in lipid metabolism. Bacterial genera of particular importance were Prevotella, Bacteroides, Clostridium, Faecalibacterium
Hu^22^	Case–control	78 (59/19)	Stool	60 (76.9%)	24.4±2.7 /24.2±4.3	—	43 (55.1%)	18 (23%)	—	The gut microbiota links to CAD progression and can potentially be used to diagnose CAD prevalence. However, the microbial taxa associated with CAD are not always reproducible across different cohorts, and only the reduced Bacteroides is a reliable indicator for CAD prevalence
Kwun^23^	Case–control	42 (22/20)	Oral swab and Stool	38 (90.5%)	—	Check	13 (59.1%)	6 (27.3%)	—	The results indicate that the relative abundance of the gut and oral microbiomes was correlated with that of the thrombus microbiome
Li^24^	Case–control	206 (126/80)	Blood	101 (49%)	23.7±3.5 ; 23.5±3.6	—	108 (52.4%)	42 (20.4%)	—	The members of family Pseudomonadacee were more frequently identified in the CAD group than the non-CAD group
Liu^26^	Case–control	20 (10/10)	Blood and Stool	14 (70%)	22.55±0.83 / 22.91±0.48/	—	—	—	—	These results indicated that the unstable angina pectoris patients had decreased serum IGF-1 level and imbalanced amino acids metabolism, which may be caused by the altered gut microbiota. It may provide a new therapeutic strategy for unstable angina pectoris
Liu^25^	Case–control	53 (30/23)	Blood and Stool	37 (69.8%)	26.94±5.33 / 25.2±3.7	12(40)/5(21.7)	—	—	—	The Dialister genus was significantly lower and Blautia, Desulfovibrio, and Succinivibrio were significantly higher in abundance in CAD patients compared with the control group, and the changes were significantly correlated with physiological indexes, such as increased lipopolysaccharides
Sawicka-smiarowska^27^	Case–control	335 (169/166)	Blood and Stool	232 (69.3%)	30.52±5.3 / 28.46±4.73	—	220 (65.7%)	66 (19.7%)	137(82)/69(50)	There are marked differences in the gut microbiome between the CAD and control groups that may translate into different metabolisms and hence may affect the development of atherosclerosis
Toya^28^	Case–control	106 (53/53)	Stool	6 gf 2 (58.5%)	29.4±5.9 / 29.1±5.7	28(52.8)/23(43.4)	51 (48.1%)	18 (16.9%)	23(45.1)/39(73.6)	CAD patients have a distinct gut microbiome compared to control patients, with reduced complement of specific butyrate- and acetate-producing gut microbes and increased relative abundance of specific inflammatory polysaccharide-producing gut microbe in advanced CAD patients compared to controls
Toya^29^	Cross-sectional	202 (88/114)	Blood and Stool	113 (55.9%)	31.2±5.9 / 27.4±5.8	81(92%)/107(94%)	78 (38.6%)	25 (12.4%)	—	There is a strong correlation between dysbiosis and mobilization of osteocalcin expressing Endothelial progenitor cell and increase release of inflammatory mediators specially in the patients with diabetes mellitus. Those reactions are related to vascular calcification and development of coronary atherosclerosis
Wan^30^	Case–control	600 (300/300)	Stool	319 (53.2%)	20.54±1.95 / 20.64±1.85	—	—	—	—	The idea of usage of gut microbiota as a diagnostic model to detect atherosclerosis and CAD is very effective. The microbiome may serve as a biomarker to predict cardiovascular disease
Yoshida 2018	Case–control	60 (30/30)	Stool	50 (83.3%)	25.1±2.8 / 24.8±4.1	27(90%)/25(83.3%)	49 (81.7%)	23 (38.3%)	18(60)/28(93)	Translational research findings identify a previously unknown link between specific gut bacteria and atherosclerosis Treatment with live B. vulgatus and B. dorei may help prevent CAD

a No SD.

ACS, acute coronary syndrome; AMI, acute myocardial infarction; CAD, coronary artery disease; IGF-1, insulin-like growth factor-1; rRNA, ribosomal RNA.

## Outcomes

### Diversity indices

#### Shannon index

The pooled analysis showed no statistically significant difference between the CAD group and control group regarding the Shannon Index (MD=0.03, 95% CI=−0.13 to 0.20, *P*-value=0.69). We observed a significant heterogeneity between the studies (*P*=*0.006*, *I*²=61%). It was solved by leave-one-out test by removing Toya 2021 (*P*=0.70, *I*²=0%), and the analysis showed a statistically significant association between the CAD group and increased Shannon Index compared with the control group (MD=0.11, 95% CI=0.01–0.22, *P*-value=0.03) (Fig. 2).

Meta-regression analysis showed no correlation between MD and covariates such as BMI, hypertension, diabetes, and smoking, as reported in Supplementary Table S2 (Supplemental Digital Content 2, http://links.lww.com/MS9/A596).

#### Simpson index

The pooled analysis showed a statistically significant association between the CAD group and increased Simpson Index compared with the control group (MD=0.04, 95% CI=0.03–0.05, *P*-value >0.00001). We observed no significant heterogeneity among the studies (*P*=0.66, *I*²=0%) (Fig. 3).

Meta-regression analysis showed no correlation between MD and BMI as reported in Supplementary Table S2 (Supplemental Digital Content 2, http://links.lww.com/MS9/A596).

#### Bacillota/firmicutes

Our analysis revealed a significant association between increased Firmicutes phylum and CAD patients compared with the control group, (MD=6.31, 95% CI=1.33–11.28, *P*-value=0.01), with no significant heterogeneity observed (Fig. 4). We conducted further analyses of genera of this phylum which showed a significant association between increased Subdoligranulum, Catenibacterium, and CAD patients, while the other genera showed insignificant differences. On analysis of individual species, we detected insignificant associations between UCG-004 Lachnospiraceae, Ruminococcaceae-UCC.014, and CAD.

#### Subdoligranulum

The pooled analysis showed no statistically significant difference between the CAD group and the control group (MD=−0.03, 95% CI=−0.08 to 0.02, *P*-value=0.28). We observed a significant heterogeneity among the studies (*P*<0.00001, *I*²=100%). It was solved by leave-one-out test by removing either Kwun 2020 (*P*=0.63, *I*²=0%) or Zhu 2018 (*P*=0.64, *I*²=0%), and the analysis showed a statistically significant association between the CAD group and increased Subdoligranulum genus compared with the control group (MD=−0.06, 95% CI=−0.06 to −0.06, *P*-value <0.00001) (Fig. 10).

#### Catenibacterium

The pooled analysis showed a statistically significant association between the CAD group and the increased Catenibacterium genus compared with the control group (MD=0.09, 95% CI=0.09–0.10, *P*-value >0.00001). heterogeneity was not applicable (Fig. 11).

#### Butyricicoccus

The pooled analysis showed no statistically significant difference between the CAD group and the control group (MD=−0.00, 95% CI=−0.01 to 0.00, *P*-value=0.27). We observed a significant heterogeneity between the two studies (*P*>0.00001, *I*²=100%) (Fig. 10).

#### Blautia

The pooled analysis showed no statistically significant difference between the CAD group and the control group (MD=0.07, 95% CI=−0.05 to 0.18, *P*-value=0.26). We observed a significant heterogeneity between the two studies (*P*>0.00001, *I*²=100%) (Fig. 10).

#### Dialister

The pooled analysis showed no statistically significant difference between the CAD group and the control group (MD=−0.57, 95% CI=−1.64 to 0.51, *P*-value=0.30). We observed a significant heterogeneity between the two studies (*P*>0.00001, *I*²=100%) (Fig. 10).

#### Roseburia

The pooled analysis showed no statistically significant difference between the CAD group and the control group (MD=−0.02, 95% CI=−0.03 to 0.00, *P*-value=0.11). We observed a significant heterogeneity between the studies (*P*>0.00001, *I*²=100%) that was not solved by leave-one-out test (Fig. 10).

#### Ruminococcus

The pooled analysis showed no statistically significant difference between the CAD group and the control group (MD=−0.11, 95% CI=−0.34 to 0.11, *P*-value=0.33). We observed a significant heterogeneity between the two studies (*P*>0.00001, *I*²=100%) (Fig. 10).

#### Enterococcus

The pooled analysis showed no statistically significant difference between the CAD group and the control group (MD=−0.02, 95% CI=−0.08 to 0.04, *P*-value=0.52). We observed a significant heterogeneity between the two studies (*P*>0.00001, *I*²=99%) (Fig. 10).

#### Eubacterium

The pooled analysis showed no statistically significant difference between the CAD group and the control group (MD=−0.26, 95% CI=−0.77 to 0.25, *P*-value=0.32). We observed a significant heterogeneity between the two studies (*P*>0.00001, *I*²=100%) (Fig. 10).

#### Flavonifractor

The pooled analysis showed no statistically significant difference between the CAD group and the control group (MD=0.01, 95% CI=−0.01 to 0.02, *P*-value=0.34). We observed a significant heterogeneity between the two studies (*P*>0.00001, *I*² =100%) (Fig. 11).

#### Holdemanella

The pooled analysis showed no statistically significant difference between the CAD group and the control group (MD=−0.03, 95% CI=−0.09 to 0.04, *P*-value=0.41). We observed a significant heterogeneity between the two studies (*P*>0.00001, *I*²=100%) (Fig. 11).

#### Lachnospira

The pooled analysis showed no statistically significant difference between the CAD group and the control group (MD=0.00, 95% CI=−0.01 to 0.01, *P*-value=0.42). We observed a significant heterogeneity between the two studies (*P*>0.00001, *I*²=100%) (Fig. 11).

#### Lactococcus

The pooled analysis showed no statistically significant difference between the CAD group and the control group (MD=0.03, 95% CI=−0.02 to 0.07, *P*-value=0.29). We observed a significant heterogeneity between the two studies (*P*>0.00001, *I*²=100%) (Fig. 11).

#### Weissella

The pooled analysis showed no statistically significant difference between the CAD group and the control group (MD=−0.01, 95% CI=−0.04 to 0.02, *P*-value=0.39). We observed a significant heterogeneity between the two studies (*P*>0.00001, *I*²=100%) (Fig. 11).

#### Lactobacillus

The pooled analysis showed no statistically significant difference between the CAD group and the control group (MD=0.41, 95% CI=−0.79 to 1.62, *P*-value=0.50). We observed a significant heterogeneity between the studies (*P*>0.00001, *I*²=100%) that was not solved by leave-one-out test (Fig. 9).

#### Clostridium

The pooled analysis showed no statistically significant difference between the CAD group and the control group (MD=−0.01, 95% CI=−0.33 to 0.30, *P*-value=0.93). We observed a significant heterogeneity among the studies (*P*<0.00001, *I*²=100%). It was reduced by leave-one-out test by removing Kwun 2020 (*P*=0.06, *I*²=71%), and the analysis showed no statistically significant difference between the CAD group and the control group (MD=−0.49, 95% CI=−1.52 to 0.54, *P*-value=0.35) (Fig. 9).

#### Faecalibacterium

The pooled analysis showed no statistically significant difference between the CAD group and the control group (MD=0.34, 95% CI=−1.57 to 2.26, *P*-value=0.73). We observed a significant heterogeneity among the studies (*P*<0.00001, *I*²=100%). It was reduced by leave-one-out test by removing Kwun 2020 (*P*=0.06, *I*²=72%), and the analysis showed no statistically significant difference between the CAD group and the control group (MD=−2.08, 95% CI=−7.28 to 3.11, *P*-value=0.43) (Fig. 9).

#### Phascolarctobacterium

The pooled analysis showed no statistically significant difference between the CAD group and the control group (MD=−0.10, 95% CI=−0.26 to 0.06, *P*-value=0.21). We observed a significant heterogeneity between the studies (*P*>0.00001, *I*²=97%) that was not solved by leave-one-out test (Fig. 9).

#### UCG-004 lachnospiraceae

The pooled analysis showed no statistically significant difference between the CAD group and the control group (MD=−0.00, 95% CI=−0.00 to 0.00, *P*-value=0.28). We observed a significant heterogeneity between the two studies (*P*>0.00001, *I*²=100%) (Fig. 12).

#### Ruminococcaceae_UCC.014

The pooled analysis showed no statistically significant difference between the CAD group and the control group (MD=0.00, 95% CI=−0.02 to 0.02, *P*-value=0.98). We observed a significant heterogeneity between the two studies (*P*=0.04, *I*²=77%) (Fig. 12).

#### Proteobacteria/pseudomonata

Our analysis revealed an insignificant association between this phylum of bacteria and CAD, (MD=−0.55, 95% CI=−7.40 to 6.29, *P*-value=0.87), with a significant heterogeneity (*P*=0.003, *I*²=89%) (Fig. 5). On further analysis of the Gammaproteobacteria class, Enterobacteriales order, and genera of this phylum, we detected a significant difference in Pseudomonas genus and CAD, while Esherichia and Shigella showed insignificant results.

#### Gammaproteobacteria

The pooled analysis showed no statistically significant difference between the CAD group and the control group (MD=1.75, 95% CI=−0.24 to 3.73, *P*-value=0.08). We observed no significant heterogeneity between the two studies (*P*=0.29, *I*²=9%) (Fig. 6).

#### Enterobacteriales

The pooled analysis showed no statistically significant difference between the CAD group and the control group (MD=1.17, 95% CI=−1.60 to 3.94, *P*-value=0.41). We observed a significant heterogeneity between the two studies (*P*=0.03, *I*²=80%) (Fig. 8).

#### Escherichia–shigella

The pooled analysis showed no statistically significant difference between the CAD group and the control group (MD=0.00, 95% CI=−0.06 to 0.07, *P*-value=0.94). We observed a significant heterogeneity between the two studies (*P*>0.00001, *I*²=99%) (Fig. 9).

#### Pseudomonas

The pooled analysis showed a statistically significant association between the CAD group and the increased Pseudomonas genus compared with the control group (MD=0.54, 95% CI=0.29–0.78, *P*-value >0.00001). We observed a significant heterogeneity among the studies (*P*>0.00001, *I*²=100%) that was not solved by leave-one-out test (Fig. 11).

#### Bacteroidetes

Our analysis showed a significant association between the Bacteroidetes order of this phylum and CAD patients (MD=−3.96, 95% CI=−7.27 to −0.65, *P*-value=0.02), with no significant heterogeneity as shown in Figure 5. On further analysis of genera of this order, we detected a significant increase in Bacteroides, Prevotella, and Alistipes, while other genera showed insignificant differences. On the other hand, we conducted an analysis of *Bacteroides vulgatus* and *Bacteroides dorei*, which showed that CAD patients have significantly decreased levels of these species.

#### Bacteroides

The pooled analysis showed no statistically significant difference between the CAD group and the control group (MD=−3.95, 95% CI=−15.67 to 7.76, *P*-value=0.51). We observed a significant heterogeneity between the studies (*P*>0.00001, *I*²=94%) was solved by the leave-one-out test by removing Kwun 2020 (*P*=0.16, *I*²=43%), and the analysis showed a statistically significant association between the CAD group and increased Bacteroides genus compared with the control group (MD=−8.60, 95% CI=−15.20 to −2.00, *P*-value =0.01) (Fig. 9).

#### Prevotella

The pooled analysis showed no statistically significant difference between the CAD group and the control group (MD=5.64, 95% CI=−12.63 to 23.92, *P*-value=0.55). We observed a significant heterogeneity among the studies (*P*<0.00001, *I*²=98%). It was solved by leave-one-out test by removing Kwun 2020 (*P*=0.14, *I*²=54%), and the analysis showed a statistically significant association between the CAD group and increased Prevotella genus compared with the control group (MD=13.27, 95% CI=4.12–22.42, *P*-value =0.004) (Fig. 9).

#### Alistipes

The pooled analysis showed a statistically significant association between the CAD group and increased Alistipes genus compared with the control group (MD=2.64, 95% CI=2.32–2.96, *P*-value >0.0001). We observed no significant heterogeneity between the two studies (*P*=0.24, *I*²=28%) (Fig. 9).

#### *B. vulgatus* and *B. dorei*

The pooled analysis showed a statistically significant association between the CAD group and decreased *B. vulgatus* and *B. dorei* species compared with the control group (MD=−10.31, 95% CI=−14.78 to −5.84, *P*-value >0.00001). We observed no significant heterogeneity between the two studies (*P*=0.18, *I*²=44%) (Fig. 12).

### Actinomycetota

We conducted a pooled analysis on the genus Actinomyces, which belongs to the phylum Actinomycetota; our results showed no statistically significant difference between the CAD group and the control group (MD=0.00, 95% CI=−0.00 to 0.00, *P*-value=0.70). We observed a significant heterogeneity between the two studies (*P*>0.00001, *I*²=99%) (Fig. 11).

### Sensitivity and specificity (ROC) of gut microbiota in CAD

**Figure 13:**
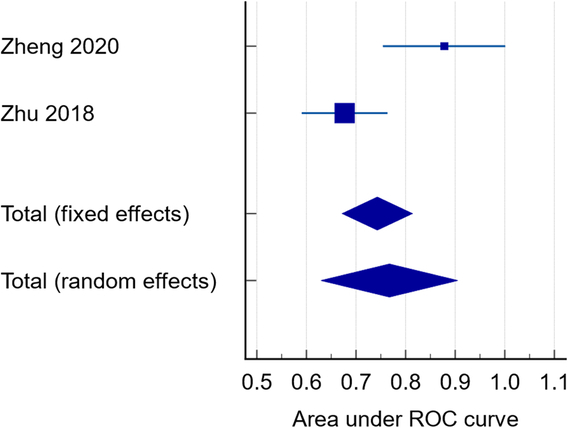
Sensitivity and specificity (ROC).

Our pooled analysis showed that the overall receiver operating characteristic (ROC) area for gut microbiota as an indicative marker in CAD was 0.767, SE was 0.0703, 95% CI (0.630–0.905). We detected significant heterogeneity among the two included studies (*P*<0.0089, *I*²=85.37%) (Fig. 13).

### Quality assessment

Individual appraisal of included studies is reported in Table 2. Only one study was rated as low quality due to inadequate definition of case and controls, while the other 17 were given a score of 7 or higher deeming them high-quality studies. As shown in Supplementary Figure S1 (Supplemental Digital Content 2, http://links.lww.com/MS9/A596), there was no evidence suggestive of publication bias for the Shannon Index; the funnel plot showed a symmetrical distribution of similar-weight studies with convergence toward the pooled treatment effect size as weights increased. Egger’s regression test was also performed and showed no evidence suggesting significant publication bias Supplementary Table S1 (Supplemental Digital Content 2, http://links.lww.com/MS9/A596).

**Table 2:** Quality assessment of the included studies.

References	Selection				Comparability	Exposure			Total points
	Is this case definition adequate ?	Representativeness of the cases	Selection of controls	Definition of controls		Ascertainment of exposure	Same method	Nonresponse rate	
Yoshida^32^	0	1	1	0	0	1	1	1	5
Zheng^33^	1	1	1	1	0	1	1	1	7
Zhu^34^	1	1	1	1	0	1	1	1	7
Chiu^17^	1	1	0	0	2	1	1	1	7
Choroszy 2022	1	1	0	0	2	1	1	1	7
Emoto 2017	1	1	0	1	2	1	1	1	8
Gao^20^	1	1	1	1	0	1	1	1	7
Gozd-Barszczewska^21^	1	1	1	1	2	1	1	1	9
Hu^22^	1	1	1	1	2	1	1	1	9
Kwun^23^	1	1	1	1	2	1	1	1	9
Li^24^	1	1	1	1	2	1	1	1	9
Liu^26^	1	1	1	1	2	1	1	1	9
Liu^25^	1	1	1	1	0	1	1	1	7
Sawicka-smiarowska^27^	1	1	1	1	2	1	1	1	9
Toya^28^	1	1	1	1	2	1	1	1	9
Toya^29^	1	1	1	1	2	1	1	1	8
Wan^30^	1	1	1	1	1	1	1	1	8
Yoshida 2018	1	1	0	1	2	1	1	1	8

## Discussion

Our results revealed a statistically significant difference in the Simpson index and Shannon between CAD patients and healthy controls. Moreover, our analysis revealed a significant association between increased Firmicutes, Proteobacteria phyla, and CAD patients compared with the control group. We conducted further analyses of the genera of these phyla which showed a significant association between increased Subdoligranulum, Catenibacterium, Pseudomonas, and CAD patients, while the other genera showed insignificant differences. On analysis of individual species, we detected insignificant associations between UCG-004 Lachnospiraceae, Ruminococcaceae-UCC.014, and CAD. Lastly, our analysis of Bacteroidales order showed a significant association with CAD; with a significant difference detected in Prevotella, Alistipes, *B. vulgatus*, and *B. dorei* among CAD patients compared with the healthy controls.

The difference in the mean of the Shannon index and Simpson reinforces the fact that the intestinal microbiome in CAD patients was more diverse compared with the healthy controls^39^. A univariate meta-regression analysis showed no correlation between previously mentioned diversity indices and BMI, hypertension, diabetes, or smoking. Although dietary and lifestyle differences may have accounted towards heterogeneity between studies and with evidence that dietary fiber, as well autoimmune disease may potentially play a role in alteration of intestinal microbiome^40–43^, and our results indicate that these factors did not contribute the mean difference between CAD and non-CAD patients.

Regarding the Firmicutes phylum, there was a significant increase in this phylum among CAD patients. This comes in line with the findings of Chen *et al*.^44^, which showed that Firmicutes abundance is higher among CAD. In addition to that, several studies have reported a positive correlation between obesity and an increased abundance of firmicutes in the intestinal microbiome of mice and humans^45–50^. On the contrary, Han *et al*.^51^ have detected a lower prevalence of Firmicutes among acute myocardial infarction patients.

Our results showed a statistically significant association between the CAD group and increased Catenibacterium genus; a positive correlation between Catenibacterium enrichment and increased dietary level of animal fat was reported^52^; these factors are major risk factors for CAD. On the other hand, there was a statistically significant decrease in *B.vulgatus* and *B.dorei* in CAD patients compared with the control group. These bacteria have been reported to have a protective function against atherosclerosis by improving endotoxemia which suggests a protective role of these species and their potential use for the prevention of CAD^31^. Our analysis has shown an insignificant difference in Lactobacillus abundance between CAD and healthy controls; this contradicts the findings of Yamashita *et al*.^53^, which have shown a statistically significant higher abundance of Lactobacillus in the CAD group.

According to our results, there was a statistically significant association between the CAD group and increased Prevotella genus compared with the control group. Prevotella genus was found to be the most common microorganism in coronary arteries atherosclerotic plaques^54^ and it was abundant among patients suffering from carotid^44^. Moreover, studies have shown that Prevotella contributes to dysbiosis in hypertensive and prehypertensive patients^55^ and cardiac valve calcification^56^. Yamashita *et al*.^53^ oppose our findings; as they reported significantly lower levels of Bacteroidetes including Prevotella among CAD patients, suggesting a protective role of the Bacteroidetes in CAD.

Our pooled analysis showed a statistically significant association between the CAD group and increased Alistipes genus compared with the control group. This reinforces the findings of Le Roy *et al*.^57^, which showed that the Alistipes genus is positively correlated with the plasma cholesterol levels in mice; hypercholesterolemia is a known risk factor for CAD. Nevertheless, Jie *et al*.^58^ analyzed the stool samples of atherosclerotic patients, and their results showed a decreased abundance of Alistipes.

Pseudomonata phylum has been previously linked to heart failure^59^, transient ischemic attacks, and stroke^60^. Notwithstanding, our study shows no statistically significant association between CAD and Proteobacteria. We conducted further analyses based on bacteria of this phylum, class Gammaproteobacteria showed an insignificant difference between CAD and controls. This opposes the findings of Almond *et al.* that demonstrated a significantly higher abundance of this class among acute coronary syndrome patients^61^. On the other side, our analysis yielded a statistically significant association between the CAD group and increased Pseudomonas genus compared with the control group. Studies have shown that Pseudomonas is a producer of trimethylamine (TMA) a precursor to Trimethylamine-N-oxide (TMAO)^62^, which if present in high amounts in serum disturbs serum lipid levels and increases CVD risk in mice and humans with up to 43% increase in risk^63–68^.

In summary, different intestinal microbiota genres had different associations with CAD. This could be attributed to numerous factors. First, between study heterogeneity and differences in sampling methods. Secondly, differences in demographics such as lifestyle and dietary habbits, as reported in Table 1, could have potentially caused these conflicts. In individuals diagnosed with CAD, an imbalance in different genres of gut microbiota such as Prevotella or Alistipes disrupts the normal microbial environment, resulting in increased concentrations of bacterial metabolites circulating in the bloodstream. These metabolites contribute significantly to the inflammation of blood vessel linings, which accelerates the progression of atherosclerosis^18,54,69–78^.

### Microbiome as a marker of CAD

Our ROC curve showed a value of 0.767, which indicates that the microbiome is an acceptable discriminatory marker of CAD^70^.

### Strengths

Our study analyzed a diverse spectrum of bacteria belonging to the major four phyla of the human microbiome (Firmicutes, Pseudomonata, Bacteroidetes, and Actinomycetota). Furthermore, we included more than 20 different genera of bacteria in our analysis and studied individual species such as *B. vulgatus* and *B. dorei*. This allowed us to reach more precise results.

### Limitation

Our study has some limitations. First, this meta-analysis includes only observational data with 3 cohorts, 1 cross-sectional, and 15 case–controlled studies. Due to the absence of randomization, each observational study bears inherent selection and confounding bias linked to patients’ individual characteristics, adjustment, or specific covariate analysis was not established in any of the included studies. Secondly, the performed meta-regression analysis might not have reported accurate results due to lack of sufficient number of studies per outcome or potentially insufficient data regarding certain baseline variables, such as alcohol consumption, dietary habbit of patients in each study or prevalence of certain autoimmune diseases which may have contributed to microbiome alteration. Finally, due to the few controlled studies published, our sample size was limited to 2101 patients.

## Conclusion

Dysbiosis is an acceptable discriminatory marker of CAD. Decreased *B.dorei* and *B.vulgatus* among these patients suggests a protective role of these bacteria. Future clinical trials are necessary to investigate the potential benefit of supplementation of these bacteria in treating or preventing CAD.

## Ethical approval

Ethics approval was not required for this review and meta-analysis.

## Consent

Informed consent was not required for this review and meta-analysis.

## Source of funding

None.

## Author contribution

Y.E.D. and Y.E.Y.: conceptualization; N.A.I.F.H., A.T., and C.A.: screening; M.A.S. and O.E.: data extraction; M.D., S.S.R., and S.S.A.: statistical analysis; S.N.N., T.M., Y.E., R.N., Y.H., M.A., Y.E.D., M.D., and R.W.: writing; Y.H. and H.A.: supervision. All authors reviewed the final manuscript and approved it.

## Conflicts of interest disclosure

All authors declare no conflict of interest.

## Research registration unique identifying number (UIN)

1. Name of the registry: http://www.researchregistry.com
2. Unique identifying number or registration ID: reviewregistry1811.

Hyperlink to your specific registration (must be publicly accessible and will be checked): https://www.researchregistry.com/browse-the-registry#registryofsystematicreviewsmeta-analyses/

## Guarantor

Yusef Hazimeh.

## Data availability statement

All data are available upon reasonable request to the corresponding author.

## Provenance and peer review

My paper was not invited.

## Supplementary Material
