# Supplementary material for: Intestinal microbiome as a diagnostic marker of coronary artery disease: a systematic review and meta-analysis
Source: Ann Med Surg (Lond). 2024 Sep 4;86(10):6105–20. doi: 10.1097/MS9.0000000000002516 (PMC11444608; doi:10.1097/MS9.0000000000002516)

*Supplementary Figure S1: Funnel plot for Shannon Index*


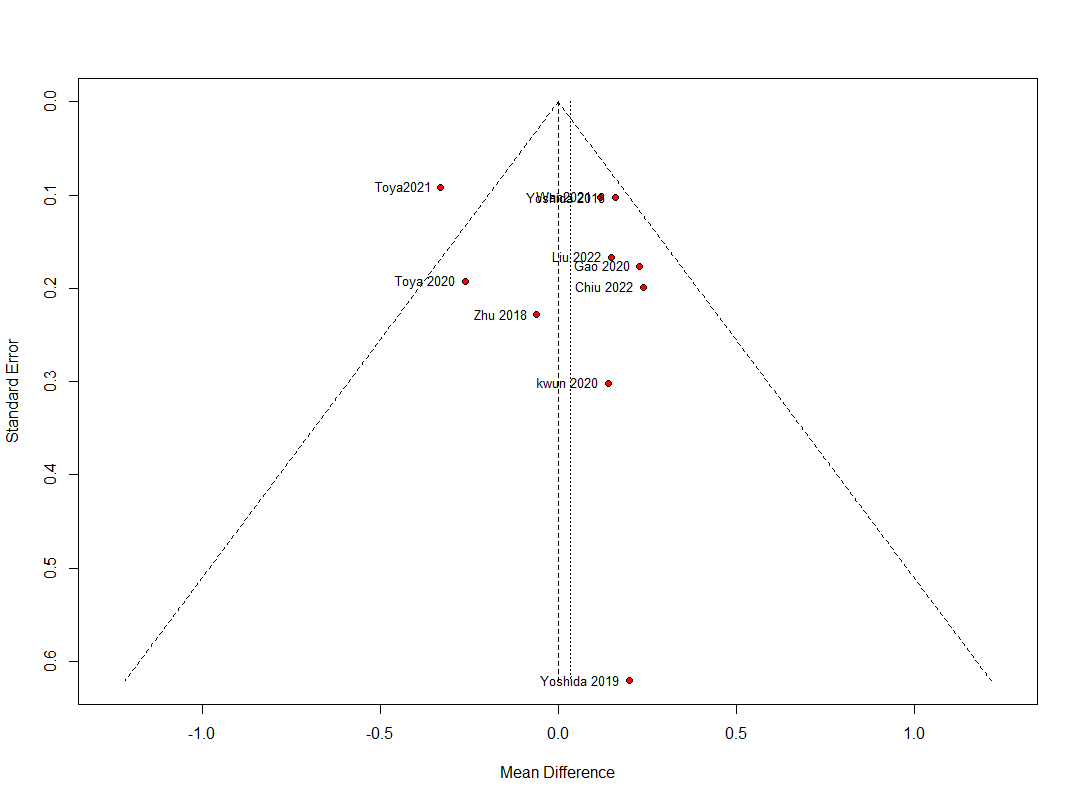


*Supplementary Table S1: Egger’s regression Test*

| **Egger’s Regression Test for Shannon Index** | | | |
| --- | --- | --- | --- |
| **Intercept** | **95% CI** | **t** | **p-value** |
| 0.95 | (-1.44; - 3.34) | 0.778 | 0.4591 |

*Supplementary Table S2. Table summarizing results of regression analysis.*

| ***Outcome*** | ***R²*** | **SE** | | **Z-value** | **Estimate** | **95% CI** | **P-value** |
| --- | --- | --- | --- | --- | --- | --- | --- |
| *Shannon Index* |  | |  |  |  |  |  |
| BMI | 41.22 | | 0.0081 | -1.6029 | -0.0129 | -0.0287, 0.0029 | 0.1090 |
| Hypertension | 0 | | 0.2960 | -0.2396 | -0.0709 | -0.6510, -0.5092 | 0.8106 |
| Diabetes | 0 | | 0.6696 | 0.0749 | 0.0502 | -1.2621, 1.3625 | 0.9403 |
| Smoking | 17.42 | | 0.01909 | -1.1366 | -0.0015 | -0.5912, 0.1572 | 0.2557 |
| *Simpson Index* |  | |  |  |  |  |  |
| BMI | 0 | | 0.0075 | -0.9063 | -0.0068 | -0.0215, 0.0079 | 0.3648 |
| Hypertension | - | | - | - | - | - | - |
| Diabetes | - | | - | - | - | - | - |
| Smoking | - | | - | - | - | - | - |

*Supplementary Figures S2: Bubble plots for Shannon Index and Simpson Index demonstrating covariate meta-regression analysis.*

Figures S2 A: Shannon Index
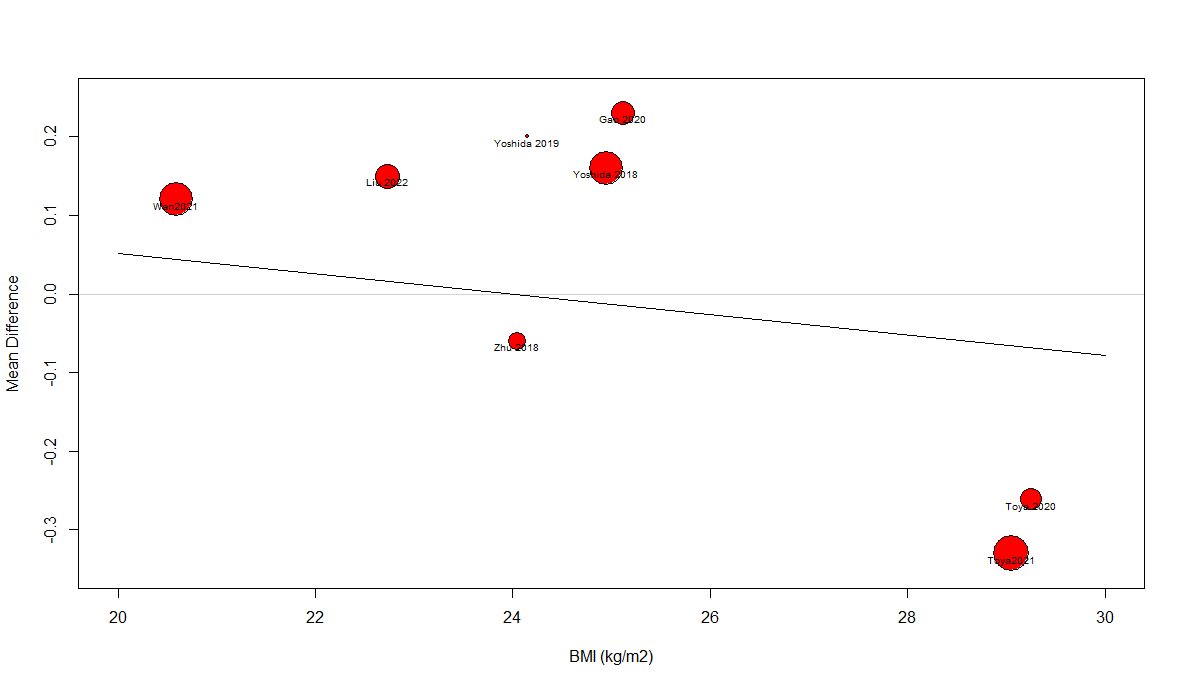

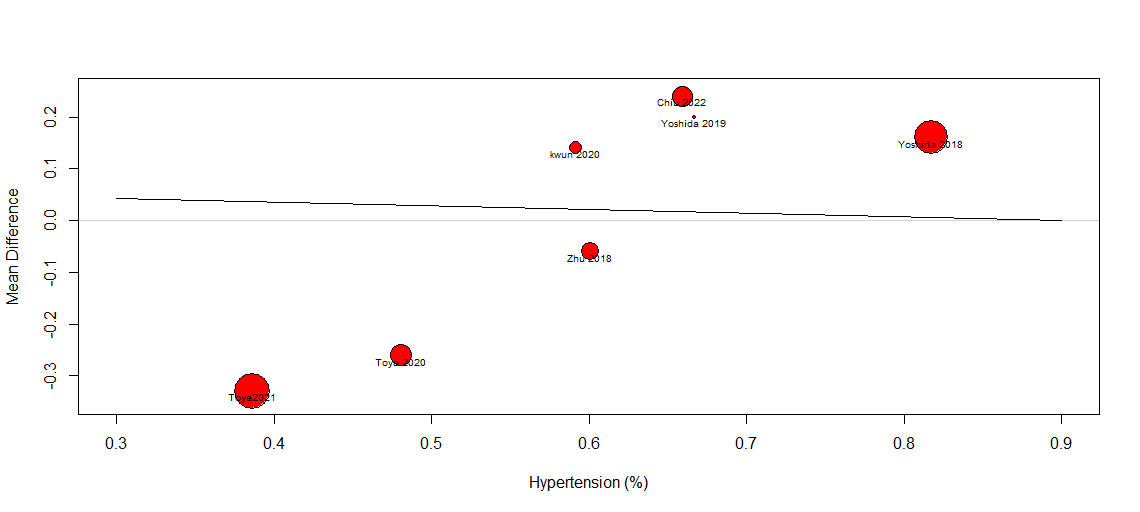

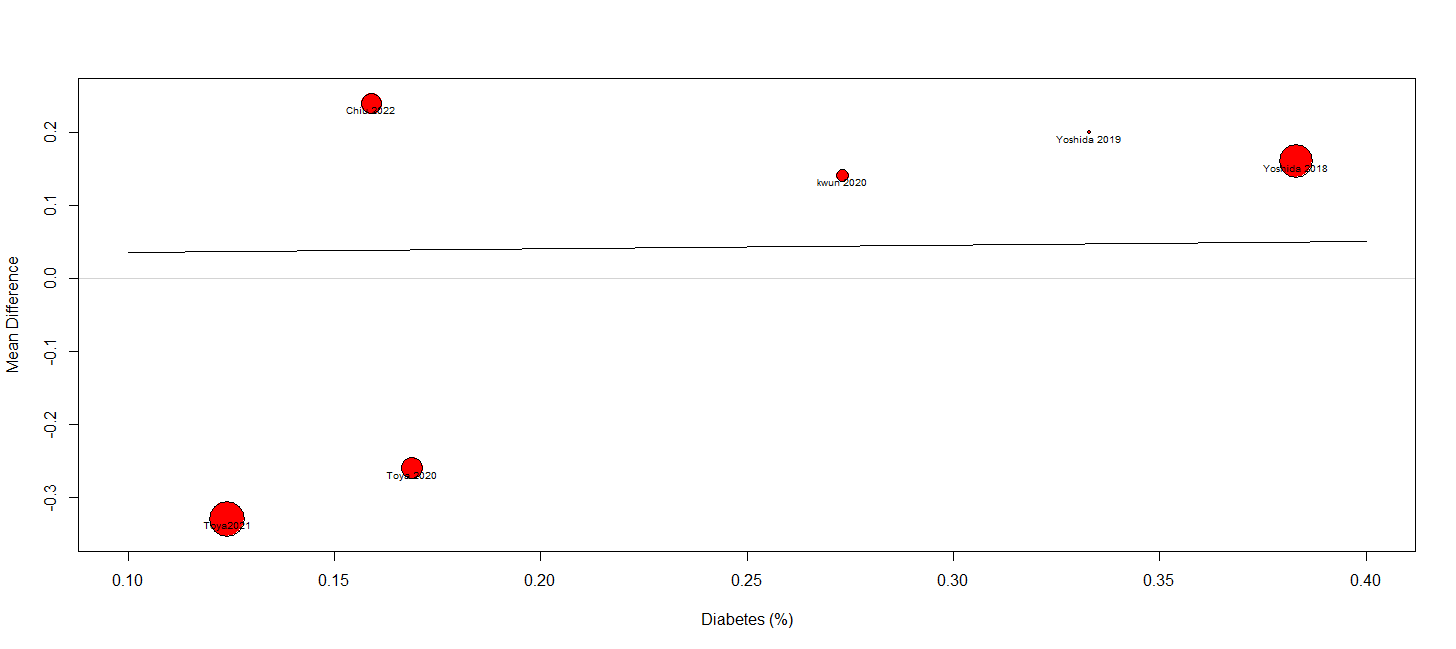

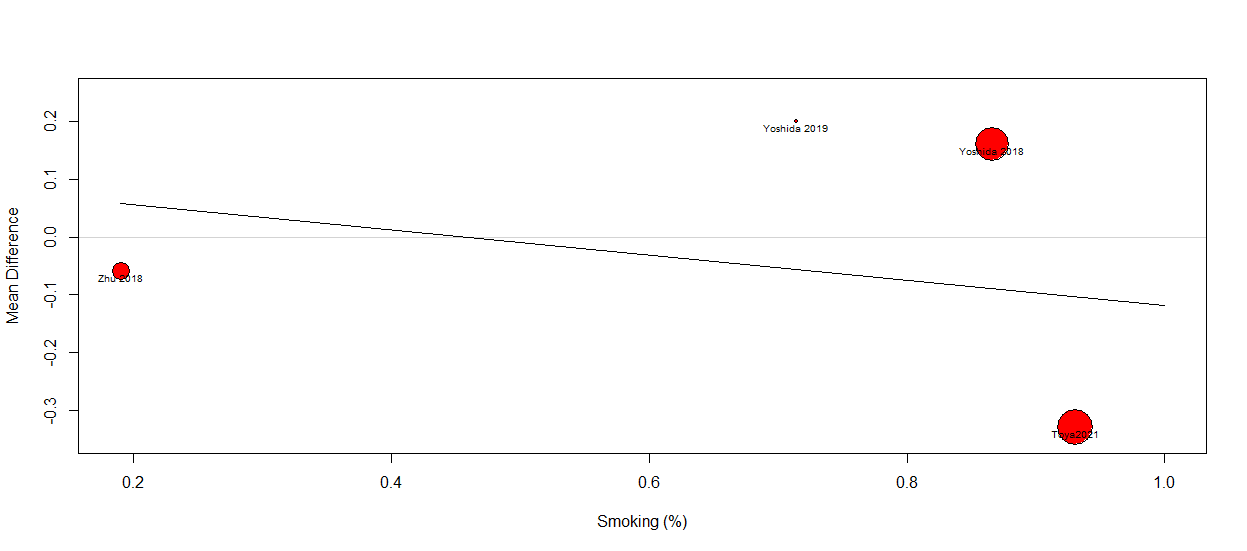


Figure S2 B: Simpson Index


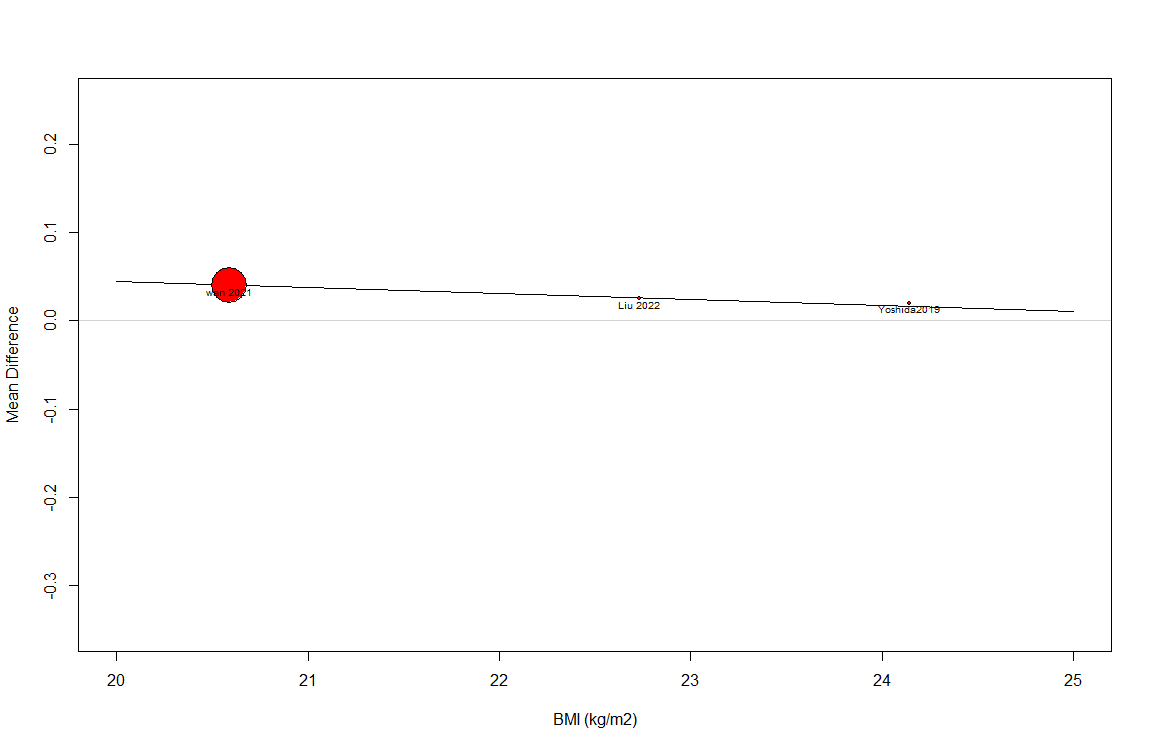

Supplement: Supplementary file 2 [file ms9-86-6105-s002.docx]
